# Analysis of prospective child development specialists’ perceptions of the hospital environment

**DOI:** 10.3389/fped.2026.1718493

**Published:** 2026-02-19

**Authors:** Güzin Yasemin Tunçay

**Affiliations:** Faculty of Health Sciences, Çankırı Karatekin University, Çankırı, Türkiye

**Keywords:** child development specialist, drawing analysis, hospital, perception, student

## Abstract

**Introduction:**

Hospitals are among the most important institutions providing healthcare services and contribute to public health through the coordination of various units. Although child development specialists also work in hospitals, this practice is generally unknown, because they are not directly involved in diagnosis and treatment processes, and therefore are not directly associated with healthcare services or the hospital environment.

**Methods:**

In the present study conducted with 30 students from the Child Development Department of Çankırı Karatekin University, Faculty of Health Sciences, changes in Child Development Department students’ perceptions of the hospital environment before and after their hospital practice were investigated. The data obtained were analyzed using thematic analysis.

**Results:**

The comparison of their pre- and post-application drawings revealed that their perceptions of the hospital environment greatly changed, and their perspectives broadened. After the internship, their tendency to view the hospital as a child development specialist's workplace differed from their pre-implementation views, but not much.

**Discussion:**

It is recommended that the theoretical course content should be further enriched with topics on hospitals, diseases, and healthcare services before the internship, and that field trips to healthcare institutions should be organized as part of the course.

## Introduction

Hospitals are among the most important institutions where healthcare services are provided to the public. They consist of closely interconnected units such as outpatient clinics, inpatient wards, intensive care units, emergency departments, blood collection units, and laboratories where illnesses are diagnosed and treated. All units work in coordination and in constant communication with each other to provide the highest quality healthcare services tailored to the patient's health condition. For example, a patient presenting to the emergency department is either discharged or referred to the appropriate unit after receiving initial treatment. Sometimes, a patient admitted to the emergency department requires emergency surgery. Whether during routine treatment or in emergencies, communication between these units and the healthcare team is crucial. This healthcare team consists of a variety of health professionals such as physicians, nurses, dietitians, physiotherapists, and psychologists. Among these healthcare professionals, another lesser-known but important group is child development specialists.

A child development specialist working in a hospital care about the developmental and psychological needs of children who are hospitalized or admitted to an outpatient clinic. Child development specialists’ role in the hospital is particularly important since they ensure that children are not negatively affected by the disease process and support the development of children who come to visit their hospitalized parents (1).

Being sick and hospitalized is a stressful situation for most individuals. Unfamiliar medical devices and sounds from these devices in an unfamiliar environment can be stressful (2). If the patient is a child, their uncertainty can be particularly intense. Children may have more difficulty coping with stressful situations like hospitalization than adults do. While adults generally understand the reason for hospitalization and the purpose of the procedures better, children, especially those younger or with developmental delays, may have difficulty comprehending this process. Furthermore, being away from their familiar home environment, family, and friends can cause them to experience feelings of fear, anxiety, and uncertainty, which may turn the process into a traumatic experience (3, 4). The level of stress experienced by a sick child can vary depending on many factors such as the quality of communication within the family, social support the family receives, the child's diagnosis, previous hospital- or hospitalization-related experiences, and parents’ coping strategies. Parents’ stress is directly reflected in their children (5). Presence of a specialist who can directly care for the child and understand their developmental characteristics can help the child cope with stress, which can significantly contribute to the child's recovery. Child development specialists both assess children's development and support them to adapt to the hospital (1, 6).

One of the places where child development specialists work is hospitals. Before they begin their professional careers, prospective child development specialists often practice in hospitals, to strengthen their theoretical knowledge through practical applications. Of them, those who lack hospital experience and cannot communicate with sick children and their parents appropriately may experience anxiety. Their pre-application personal experiences may lead to preconceptions about the hospital environment and resulting anxiety. Negative health experiences may cause them to perceive the hospital environment as a source of stress, which may lead to reluctance to perform hospital practices, to have difficulty in communicating with sick children and their parents, and to display decreased performance.

This study was aimed at investigating *Child Development Department students’* (hereafter referred to as “participants”) perceptions of the hospital environment before and after their hospital practice. A similar study was not identified in the literature review, showing that this research is original. The findings are expected to support their adaptation to the hospital environment and to shed light on issues that should be addressed before they perform their practices.

## Materials and methods

### Research model

The study employed a descriptive model of qualitative research. In the present study, the purpose was to investigate how Child Development Department students’ pre-practice perceptions of the hospital environment changed after the practice. To achieve this purpose, the same data collection form was administered to the participating students before and after they performed hospital practices.

### Study participants

The study sample consisted of 30 students who attended the Child Development Department of the Faculty of Health Sciences at Çankırı Karatekin University, took the Hospital and Child I course in the fall semester of the 2023–2024 academic year and successfully completed the Hospital and Child II practical course in the spring semester.

According to the department curriculum, students take Hospital and Child I, and Hospital and Child II courses in their third year at school, 41 students took the course. Of them, 30 participated in the study before and after the implementation. Of the participating students, while most were between the ages of 20 and 23 years, a small number were ≥24 years old. Of those who took the Hospital and Child I, and Hospital and Child II courses, the majority were third-year students, the remaining were second- and fourth-year students. Most of them did not attend a health high school and were graduates of Anatolian High Schools, where students take additional hours of foreign language courses compared to students attending regular high schools. Of them, 47% previously accompanied a patient in the hospital, and 43% were previously hospitalized. Of those who were hospitalized, the majority (62.0%) were hospitalized for three or more days. The vast majority of them (83.3%) reported that they had no illness (Table 1).

**Table 1:** Descriptive characteristics of the participants.

Characteristics		Number	%
Age (years)	20–21	13	43.3
	22–23	13	43.3
	≥24	4	13.4
Sex	Women	30	100.0
Year at school	2	1	3.0
	3	26	87.0
	4	3	10.0
Being a graduate of a health high school	Yes	2	6.7
	No	28	93.3
Type of the high school from which the participants graduated	Anatolian high school	23	77.0
	İmam Hatip high school^a^	3	10.0
	Regular high school	2	6.5
	Vocational high school	2	6.5
Having accompanied a patient in the hospital	Yes	14	47.0
	No	16	53.0
Having been hospitalized	Yes	13	43.0
	No	17	57.0
Number of days they were hospitalized (*n* = 13)	1–2 days	5	38.0
	≥3 days	8	62.0
Having an illness	Yes	5	16.7
	No	25	83.3
Having regular health check-ups (*n* = 5)	Yes	2	40.0
	No	3	60.0
TOTAL		30	100.0

a A high school where students are trained to be priests or prepared for higher education.

#### Data collection tool

The data collection form developed by the researcher includes questions on the respondents’ demographic and intervention-related characteristics such as whether they were hospitalized, and whether they want to work in a hospital. In the data collection form, they were asked to draw a picture of the hospital environment as a response to the following question: “What comes to your mind when you think of a hospital? Please, illustrate it.” The form was prepared using literature and the researcher's experience as a nurse and academic.

#### Data collection process

Before the study was conducted, ethical approval was obtained from the Çankırı Karatekin University Health Sciences Ethics Committee (Decision number: 11, Decision date: January 17, 2024). In addition, written permission was obtained from the Head of the Child Development Department on February 14, 2024, for the purpose of conducting the research. The participants were contacted personally, and the study was conducted on a voluntary basis. Some of the students did not want to participate in the study. A prerequisite for taking the Hospital and Child II course is to complete the Hospital and Child I course successfully.

Therefore, the students who participated in the study were those who had successfully completed the Hospital and Child I course. Data were collected on the first day (March 1, 2024) and the last day (May 31, 2024) of the Hospital and Child II course during the 2023–2024 Spring semester; in other words, data were collected before and after the hospital implementation. Some students who did not attend courses on those days filled out the data collection form either before or after the implementation. These students and students who did not wish to participate in the study were not included in the study; 30 out of 41 students participated in the study. The participants drew pictures on A4 paper based on the question at the end of the data collection form, “What comes to your mind when you think of a hospital? Please, illustrate it.” During the data collection process, the participants were provided with crayons and pastels to make their drawings. They were also told they could use pencils or pens if they wished, without a time limit.

#### Data analysis

The analysis of the drawings collected from the students in the study was performed based on the thematic analysis method suggested by Braun and Clarke (7). The steps involved were as follows: being familiarized with the data, creating initial codes, and searching for themes and identifying themes. For the credibility criterion, students were asked: “What comes to your mind when you think of a hospital? Please, illustrate it.” No other guidance was provided. The data were analyzed using an inductive approach, and themes were generated. Opinions of two experts who were faculty members specialized in child development and education were also obtained in the development of the themes. The themes identified by the experts from the drawings were compared with the themes obtained by the author. Then, the final themes were created. Inter-expert agreement (coders) above 70% is deemed reliable (8). It was 92%, this score was calculated using the inter-coder reliability formula (9) For the 8% disagreement in themes, a joint re-evaluation was conducted by the researcher and experts (coders). In the first stage, the themes were generated by the experts. After a one-month interval, the themes were re-generated, and the two sets of themes were compared to reach a consensus. The themes should reflect the participants’ experiences, independent of the researcher's personal opinions (7). This approach was also followed in this study. This procedure also contributed to the dependability of the study. Some students were observed depicting, especially in their second set of drawings, the physical conditions of the hospitals and family health centers where they completed their practicum. Information about the physical conditions at the practice sites became an important data source in the creation of the themes. To ensure the transferability of the study, information was provided about the students’ demographics, the data collection process, and the analysis stages. For the confirmability criterion, concrete indicators from the themes were used and evaluated independently of the researcher's personal opinions. Thus, the criteria of credibility, dependability, transferability, and confirmability (10) were met in this study.

### Limitations

The study was conducted with students who had successfully completed the Hospital and Children I course and were taking the Hospital and Children II course, and the results are limited to this sample.

## Results and discussion

The Hospital and Child I course is a theoretical course that covers basic hospital concepts and prepares students for situations they may encounter in the hospital environment before they begin their practical work. As part of the Hospital and Child I course, they also visit the practical room within the Faculty of Health Sciences whose aim is to provide students with preliminary information about the hospital environment, to familiarize them with medical devices, and to alleviate any hospital-related anxiety they may have.

In the Hospital and Child II course, they gain experience in hospitals and family health centers and put their theoretical knowledge into practice. In this course, they interact with children and their families in the hospital environment on a weekly basis, organize activities and training, and conduct observations. There are several units in the hospital where they work during their hospital practice: child development, pregnancy school, pediatric ward, obstetrics and gynecology ward, and pediatric outpatient clinic. They carry out some of their practical work within the course at the pregnancy school. In the pregnancy school, they plan training sessions for pregnant women under the supervision of the instructor.

Students also prepare educational and entertaining materials and organize bedside activities for children in the ward. Furthermore, every year, preparations are made for the April 23rd (Turkish National Sovereignty Day dedicated to children) and Spring Festival, and in addition to children in the ward, children who present to the outpatient clinic with their families, and pregnant women are also encouraged to participate in the festival. In the pediatric outpatient clinic, students also conduct observations and learn about the health problems of children admitted to the clinic. If a physician examining the child deems necessary, they refer the child together with their parents to a child development specialist. The child development specialist in the hospital meets with parents sent by the outpatient clinic, provides counseling, makes recommendations, performs preliminary diagnoses, refers the child to other units if necessary, and schedules follow-up appointments. Students observe these meetings and activities carried out by the child development specialist. At the Family Health Centers, students observe how the education is provided to families, how babies and children are vaccinated, how their height and weight are measured, and how tasks in the center are performed.

The results demonstrated that the vast majority of the participants (90.0%) considered working in a hospital. Hospital internship experiences changed the participating students’ minds, because 40% of them who initially did not consider working in the hospital changed their minds, which indicates that hospital internship experiences reshaped their perceptions of their profession and influenced their career choices. Of the students who considered working in a hospital before the internship, none changed their minds afterward (Table 2).

**Table 2:** Distribution of the participants in terms of their opinions about working in a hospital.

Opinions	Number	%
I considered working in a hospital before the hospital application and I still consider it.	15	50.0
I did not consider working in a hospital before the hospital application but now I consider it.	12	40.0
I did not consider working in a hospital before the hospital application and I still do not consider it.	3	10.0
Total	30	100.0

The analysis of the students’ drawings of the hospital practice revealed that their post-application drawings were different from their pre-application drawings. Before the application, students mostly focused on three themes in their drawings and depicted them within the hospital as follows: p*atient bed (56.6%), ıntravenously injected serum saline (53.3%), basic medical supplies such as syringes and stethoscopes (43.3%), and inside of the hospital (43.3%).* After the practice, there was a decrease in these percentages (20.0%, 20.0%, 26.6%, and 36.6%, respectively), which suggests that they had a limited perspective before they saw the hospital environment and its functioning, which changed after the hospital practice. None of the participants drew a picture of a child Development Specialist or their work environment before the practice, which was an expected result, because they had not seen a hospital before. However, although there was an increase in this theme after the practice, the proportion of related drawings was only 13.3%. There was only one public hospital in the province where the study was conducted and the number of child development specialists (sometimes two, sometimes one) working in that hospital was limited; thus, the participants had limited opportunities to observe child development specialists as health professionals and to have them as role models. Furthermore, the presence of 41 students in the hospital practice may be considered a contributing factor to this limitation. The only theme with a high percentage (83.3%) among the participants after the practice was “Reflection of Practice Experiences.” Students reflected on their experiences they gained during the practice in their drawings which manifested themselves in the activities they completed during the practice, the training they provided, and the depictions of hospital or family health center settings or individuals.

In their drawings, the participants depicted the child development specialist's room, the pregnancy school, the midwife working at the pregnancy school, the area where meetings were held on the morning of the practice, or their bedside activities as child development specialists which suggests that the hospital practice affected them significantly, and that they developed different perspectives about the hospital environment (Table 3).

**Table 3:** Distribution of the participants’ drawings of the hospital reflecting themes .

Themes	Before the application (*n* = 30)		After the application (*n* = 30)	
	Number	%	Number	%
Hospital bed	17	56.6	6	20.0
Intravenous injection of serum saline	16	53.3	6	20.0
Basic medical supplies such as syringes and stethoscopes	13	43.3	8	26.6
Inside of the hospital	13	43.3	11	36.6
Child development specialist	0	0.0	4	13.3
Hospital building	4	13.3	7	23.3
Outside of the hospital	2	6.6	3	10.0
Inside and outside of the hospital	2	6.6	8	26.6
Toys/educational materials	2	6.6	3	10.0
Happy child	1	3.3	7	23.3
Happy adult	2	6.6	3	10.0
Happy healthcare worker	2	6.6	3	10.0
Unhappy/anxious adult	5	16.6	5	16.6
Unhappy child	3	10.0	1	3.3
Anxious child	3	10.0	2	6.6
COVID-19 pandemic/medical mask	2	6.6	6	20.0
Personal experience	2	6.6	2	6.6
Reflection of application experiences	0	0.0	25	83.3
Ambulance	6	20.0	7	23.3
Medicine	0	0.0	2	6.6

There was a slight increase in the number of students who drew medical masks evoking the COVID-19 pandemic in their post-application drawings. Although the pandemic was no longer active, this increase was attributed to the fact that some healthcare workers and patients wore masks during outpatient clinic visits. Because hospital practices were conducted during the winter and spring seasons, seasonal infectious diseases were common, and therefore, free masks were available at the hospital entrance for individuals to use. This suggests that the participants did not think that wearing masks was a necessity in their daily lives; however, they felt that wearing them would protect them, especially in crowded environments like outpatient clinics, where infectious diseases are likely to be prevalent.

As presented in Table 3, there was a significant increase in the percentage of the participants drawing happy children in their post-application drawings (from 3.3% to 23.3%). This increase is attributed to their involvement in activities with the children during the intervention. For instance, they witnessed children's joyful and enjoyable moments during bedside and festive activities and reflected these moments in their drawings. The participants observed unhappy/anxious adults most of whom were probably parents and depicted them both in their pre- and post-application drawings.

**Figure 1:**
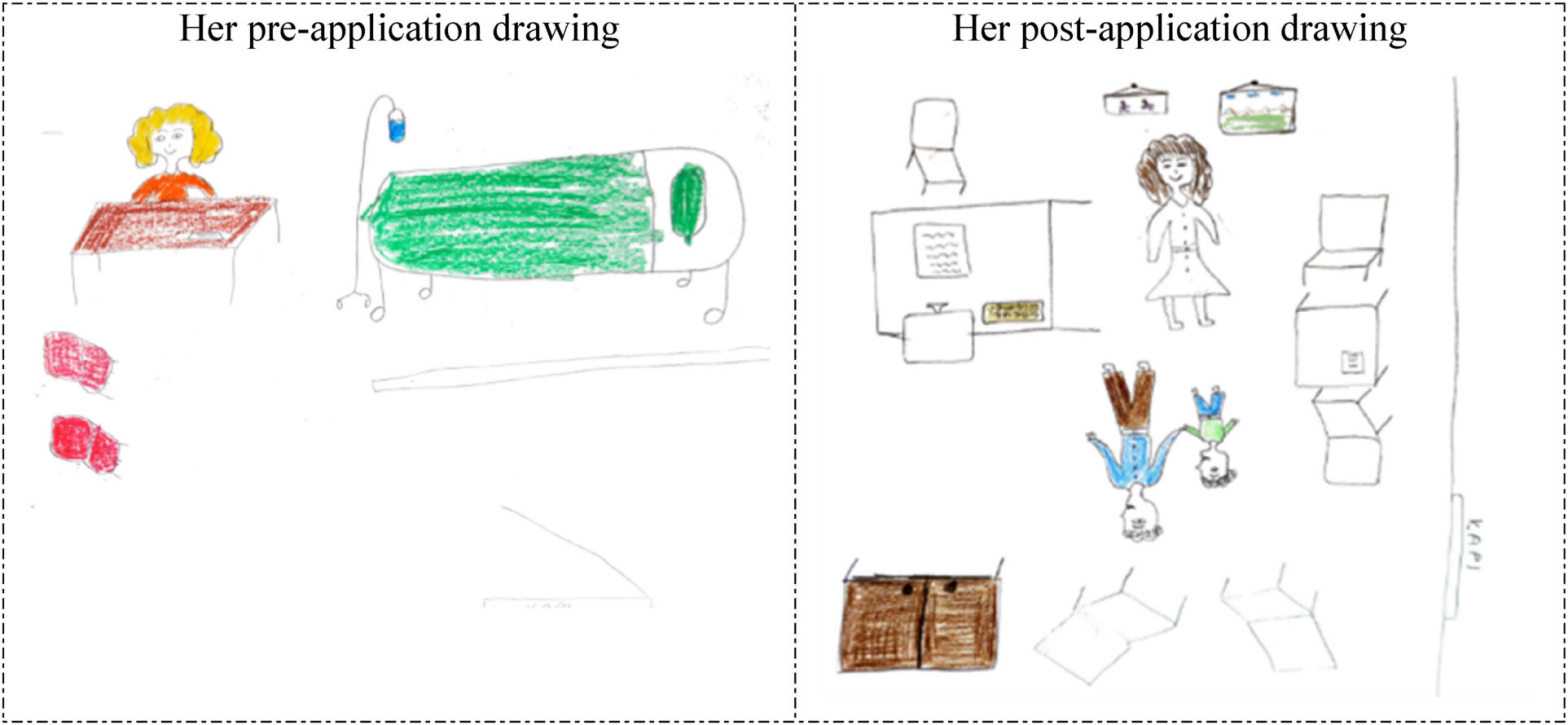
Drawings depicted by the 5th participant before and after hospital practice. The participant who previously accompanied a patient in the hospital and was previously hospitalized for 7 days, did not consider working in the hospital before the hospital practice but now wanted to work in the hospital, and stated that having regular health checks were necessary. Kapı: Door; Çocuk Gelişimci: Child development specialist.

**Figure 2:**
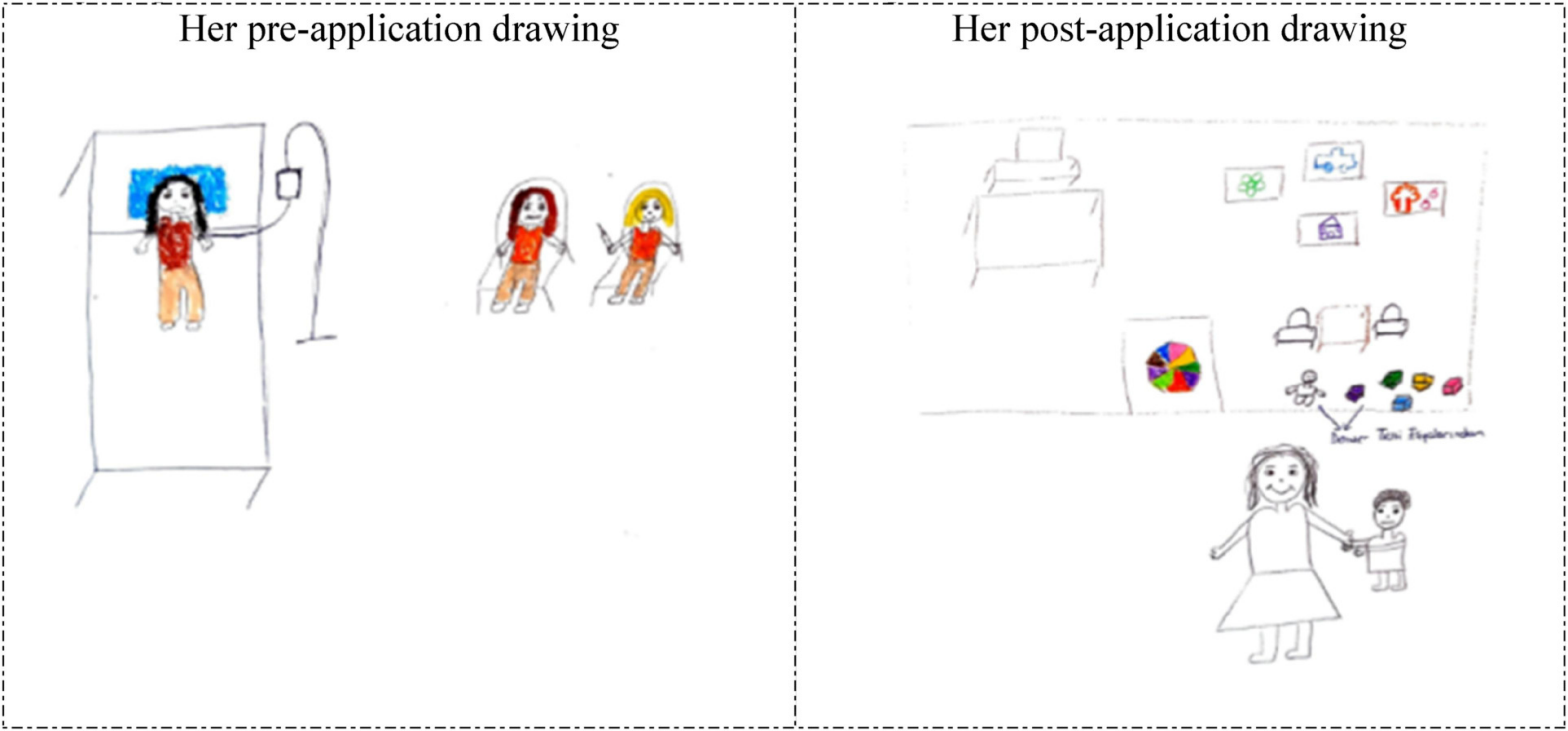
Drawings depicted by the 22th participant before and after hospital practice. The participant who previously accompanied a patient in the hospital and was previously hospitalized for 2 days, and stated that she did not consider working in the hospital before the hospital practice but now she wanted to work in the hospital. Denver testi eşyaları: Denver (Denver Developmental Screening) test materials.

**Figure 3:**
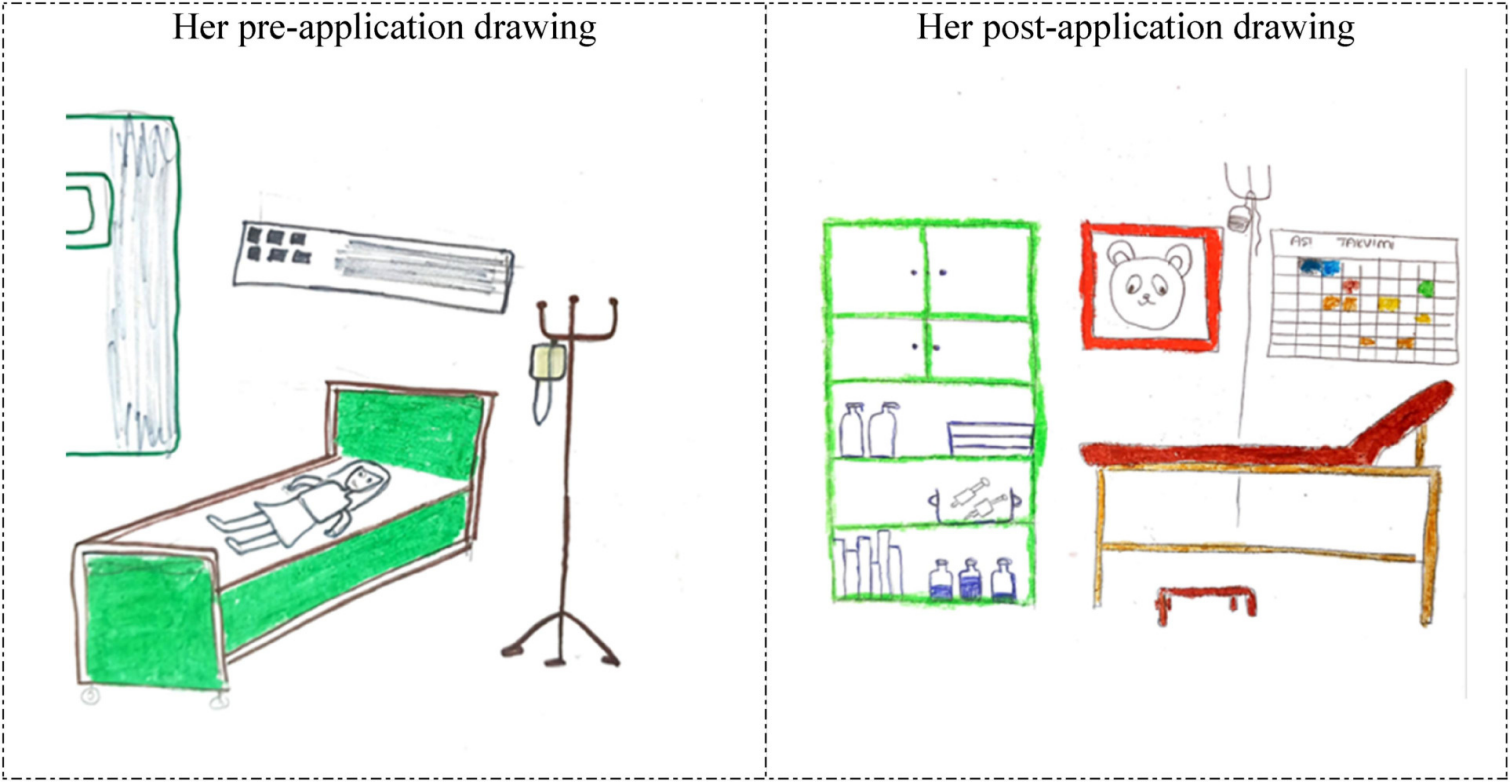
Drawings depicted by the 25th participant before and after hospital practice. The participant who previously accompanied a patient in the hospital and was previously hospitalized for 1 day stated that she did not consider working in the hospital before the hospital practice but now she wanted to work in the hospital. Aşı Takvimi: Immunization Schedule.

**Figure 4:**
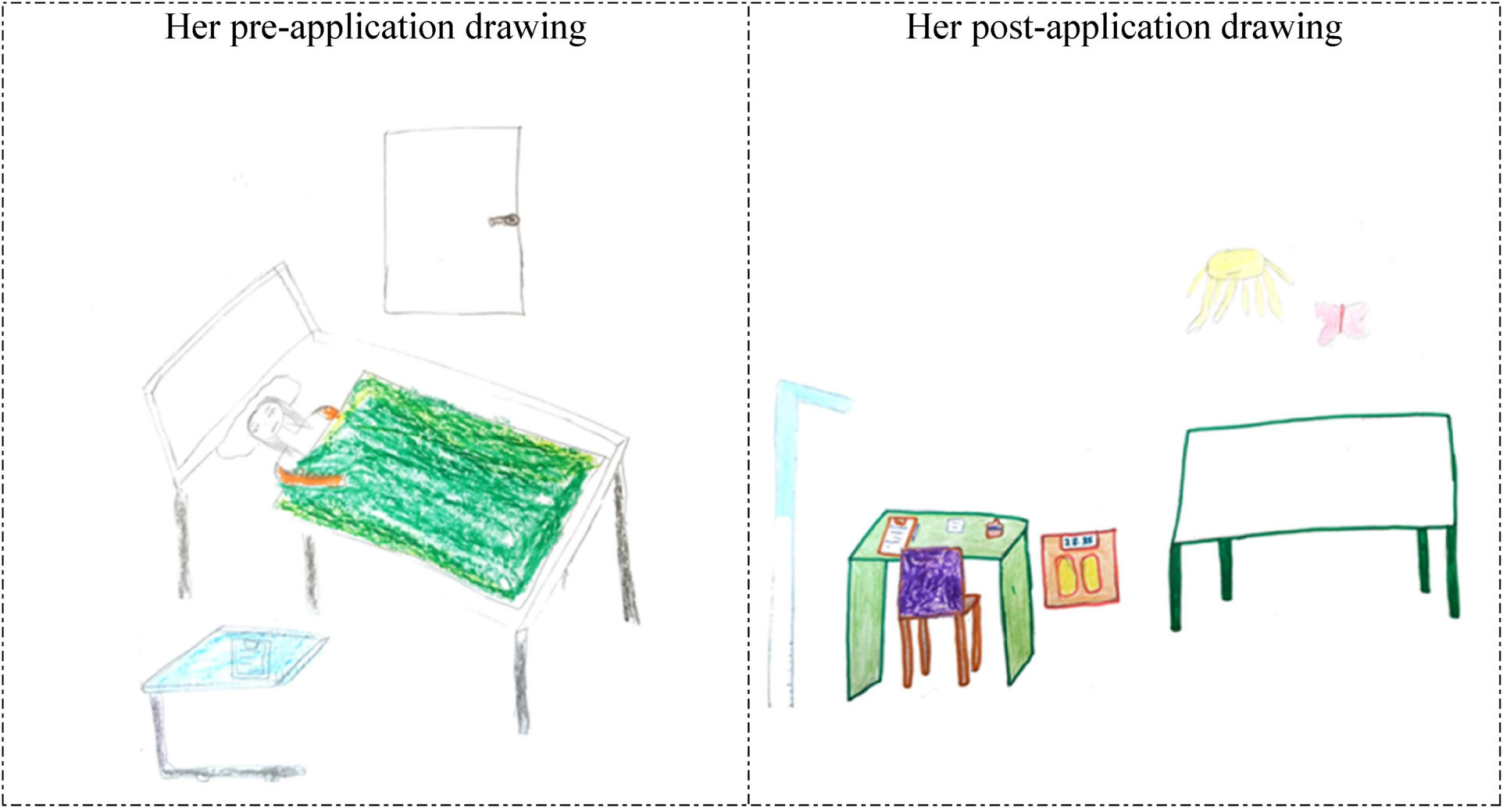
Drawings depicted by the 26th participant before and after hospital practice. The participant who previously accompanied a patient in the hospital was previously hospitalized for 1 day, considered working in the hospital before the hospital practice and still wanted to work in the hospital.

The analysis of the participants’ drawings based on their descriptive characteristics revealed that the majority (80%) of them who were previously hospitalized or who previously accompanied a patient depicted a patient bed/stretcher in their pre-application drawings. It is noteworthy that some of the drawings depicted an unhappy/anxious patient lying in bed, which suggests that the participants reflected their personal experiences in their drawings. However, these participants depicted elements related to their practice environment in their post-application drawings, demonstrating a significant change in this regard compared to their pre-application drawings. Examples of this can be viewed in the drawings below (Figures 1–4).

The 5th and 22nd participants depicted unhappy and anxious patients lying in bed in their drawings prior to the hospital practice (pre-application drawings in Figures 1, 2). However, in their post-application drawings (Figures 1, 2), they depicted a child development specialist's office, a happy mother arriving for an appointment, and a worried child.

In Figures 3, 4, the 25th and 26th participants depicted a child examination room at the Family Health Center, which demonstrates that the hospital practice positively affected their perspectives of the hospital environment from a professional perspective. The reflection of the child development specialist's office and work environment, and parents’ reactions in the students’ drawings, in particular, suggests that their perception of hospitals was shaped by their professional perspectives, indicating that the students began to perceive the hospital environment as a workspace where child development specialists fulfills their professional role. In their pre-application drawings, the 25th and 26th participants depicted a hospital room and an unhappy/anxious woman lying in bed, which suggests that they associated the hospital with illness and perceived it as an emotionally negative place. However, in their post-application drawings, the same participants depicted a pediatric examination room in a Family Health Center. Their inclusion of wall decorations and colorful details in these drawings suggests that they perceived the healthcare facility as a warmer, more child-friendly environment.

**Figure 5:**
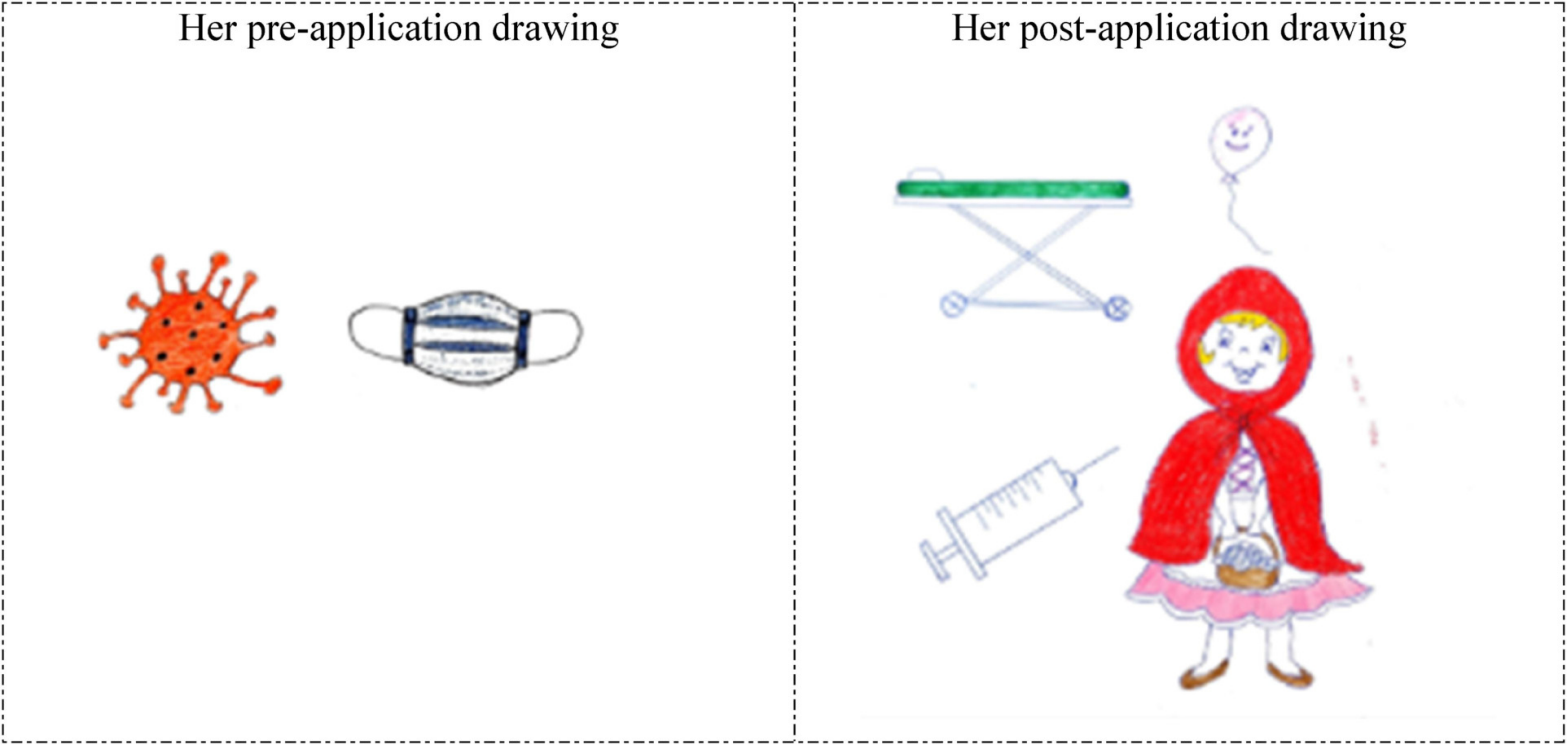
Drawings depicted by the 29th participant before and after hospital practice. The participant who had never been hospitalized, did not consider working in the hospital before the hospital practice, but now wanted to work in the hospital.

In her pre-application drawing, the 29th participant depicted a COVID-19 virus and a medical mask, reminding the COVID-19 pandemic, which suggests that the student had encountered the pandemic through the concepts of disease and hospital (Figure 5). In her post-application drawing, she directly reflected her application experiences (festival costume, festival preparations with balloons, patient stretcher/bed, and basic medical supplies such as syringes). She stated that she had not considered working in a hospital before the application, suggesting that she negatively associated the concepts of hospital and disease with the COVID-19 pandemic. On the other hand, the same participants’ starting to consider working in a hospital after the application indicates that her perception changed positively (Figure 5).

**Figure 6:**
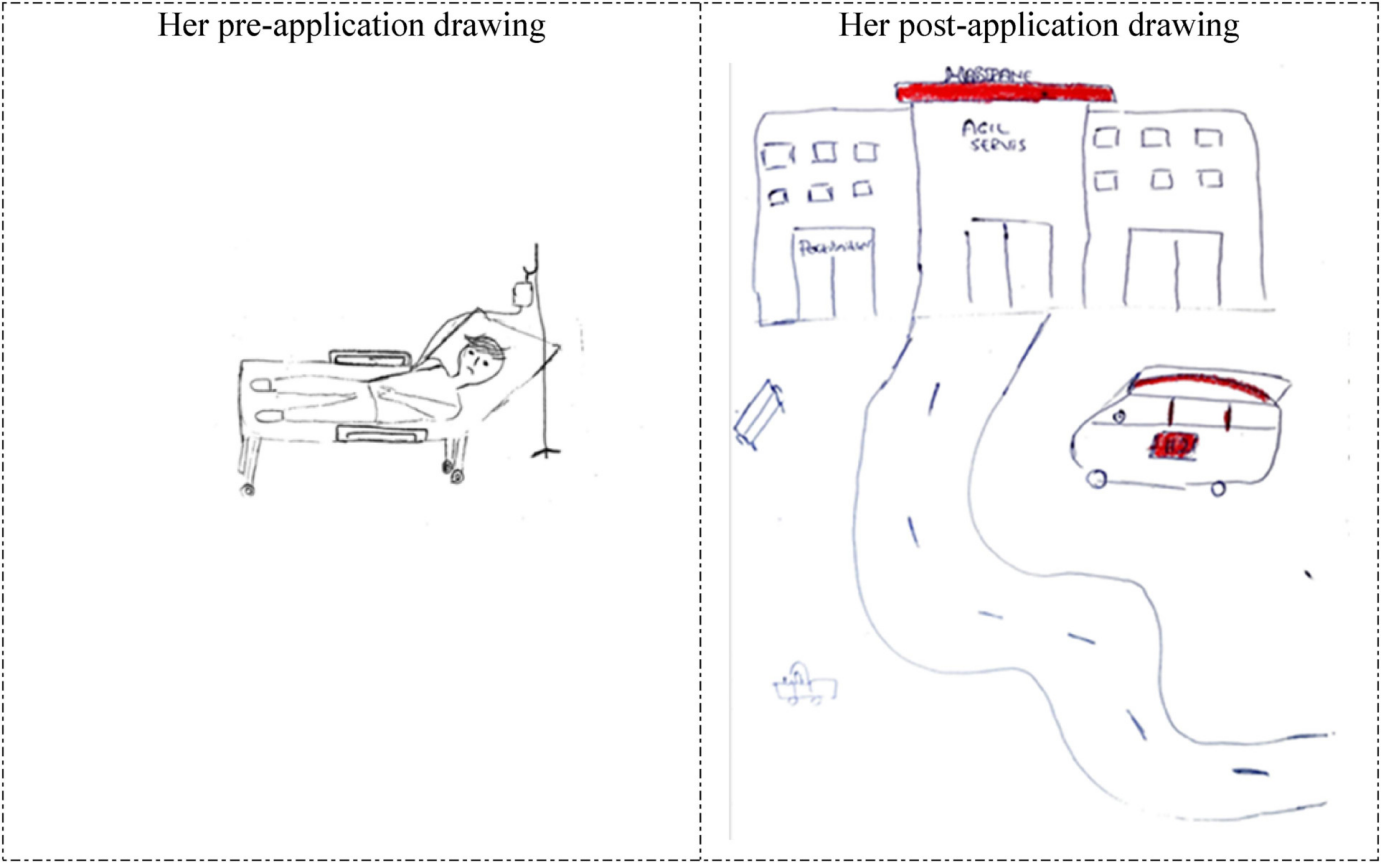
Drawings depicted by the 4th participant before and after hospital practice. The participant who graduated from a vocational school of health, did not consider working in the hospital before the hospital practice but now wanted to work in the hospital. Hastane: Hospital; Acil service: Emergency service; 112: Emergency call center.

Of the students participating in the study, two had graduated from a vocational school of health. In their pre- and post-application drawings, they both depicted hospital settings. One of them depicted an unhappy/anxious patient lying in bed in her pre-application drawing and the hospital exterior, including the emergency service in her post-application drawing (Figure 6).

**Figure 7:**
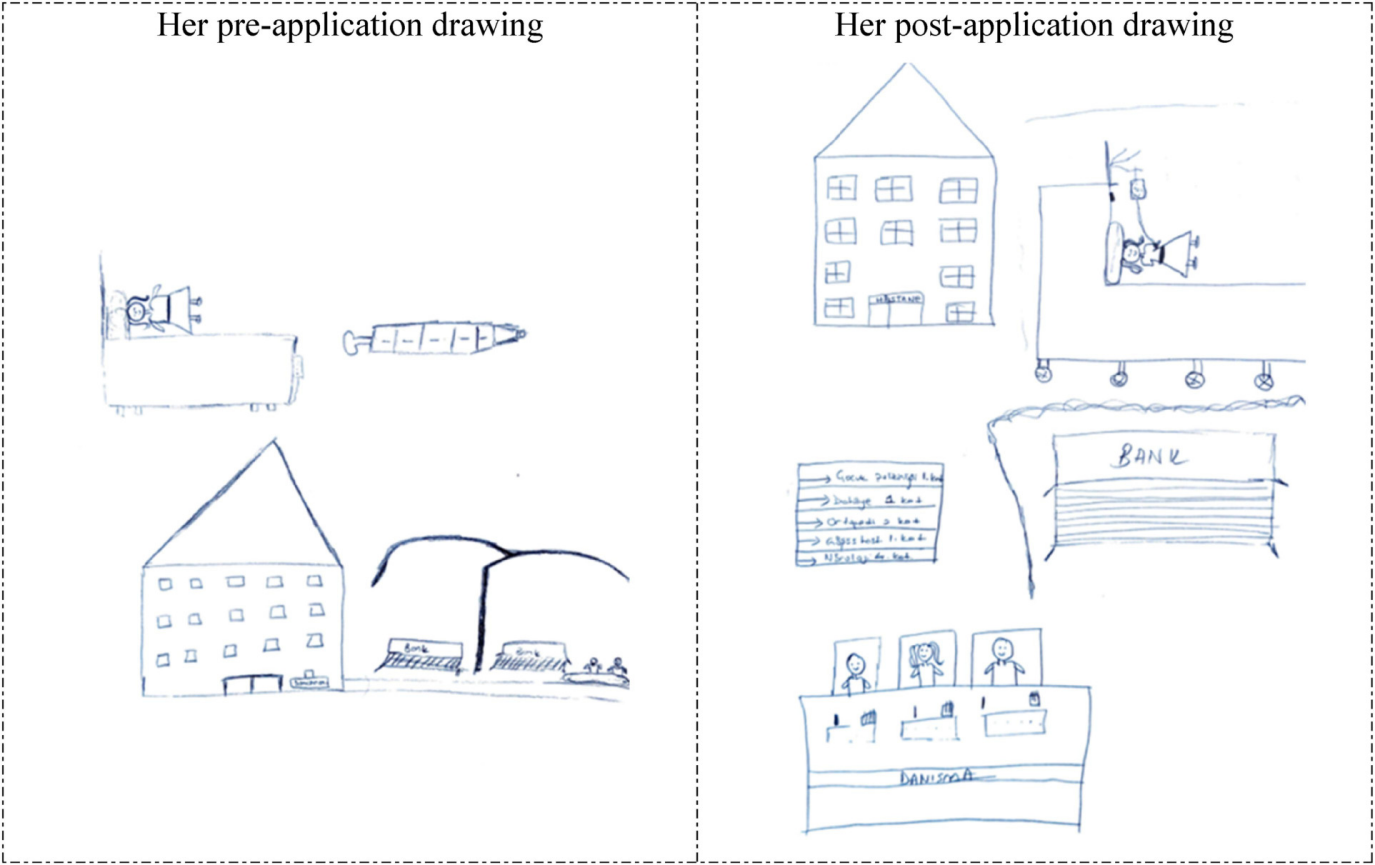
Drawings depicted by the 12th participant before and after hospital practice. The participant who graduated from a vocational school of health, did not consider working in the hospital before the hospital practice but now wanted to work in the hospital. Danışma: Information desk; Bank: Bench.

The other participant (12th participant) depicted the hospital building, a happy patient lying in bed, benches outside the hospital, and a large syringe in her pre-application drawing. In her post-application drawing, in addition to her pre-application drawing, she depicted people at the information desk at the hospital entrance and the direction signs showing which department was on which floor of the hospital (Figure 7). Neither participant's post-application drawings included any elements related to the child development profession or their practical experience in the field (e.g., child development room, pregnancy school, bedside activities, or festivals), probably due to their previous hospital practice experiences. Their having pre-formed ideas about the hospital environment based on their previous experiences suggested that the practice they performed within the scope of this course did not have a significant effect on their perception of the hospital.

**Figure 8:**
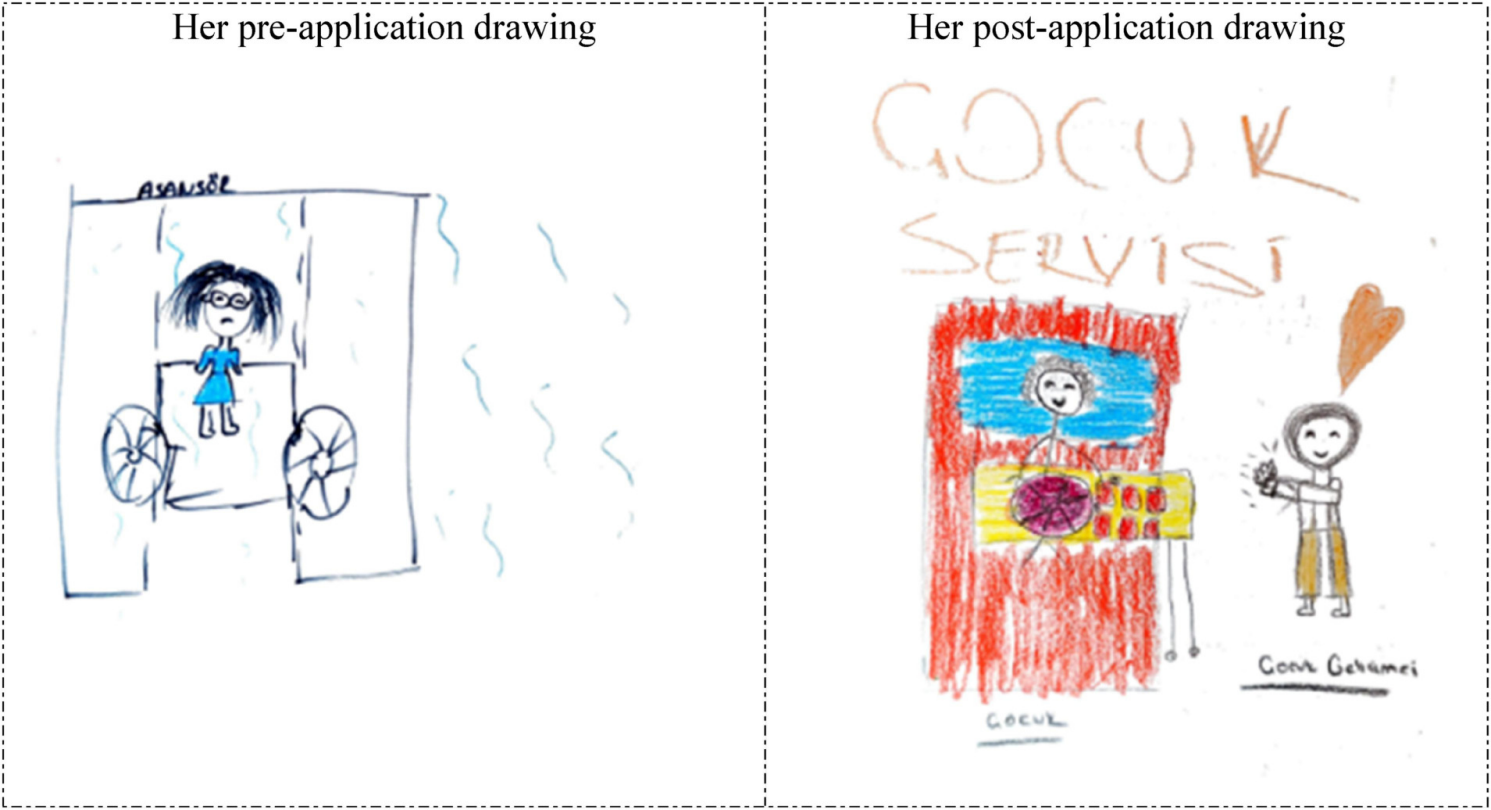
Drawings depicted by the 20th participant before and after hospital practice. The participant who previously accompanied a patient in the hospital was previously hospitalized for 1 day, considered working in the hospital before the hospital practice, still wanted to work in the hospital, and stated that having regular health checks were necessary. Asansör: Lift; Çocuk servisi: Pediatric service; Çocuk: Child; Çocuk gelişimci: Child development specialist.

The 20th participant depicted a worried woman in an elevator in her pre-application drawing and a bedside activity she performed with a hospitalized child in her post-application drawing. In her post-application drawing, she depicted herself as a `child development specialist` who applauded the child for completing the activity successfully (she drew a large heart on her head). She depicted both herself and the child as happy. In her post-application drawing, she clearly demonstrated the role of a child development specialist. This drawing differs from the other drawings because it is the only example in which the participant depicted herself and positioned herself within the role of a child development specialist (Figure 8).

**Figure 9:**
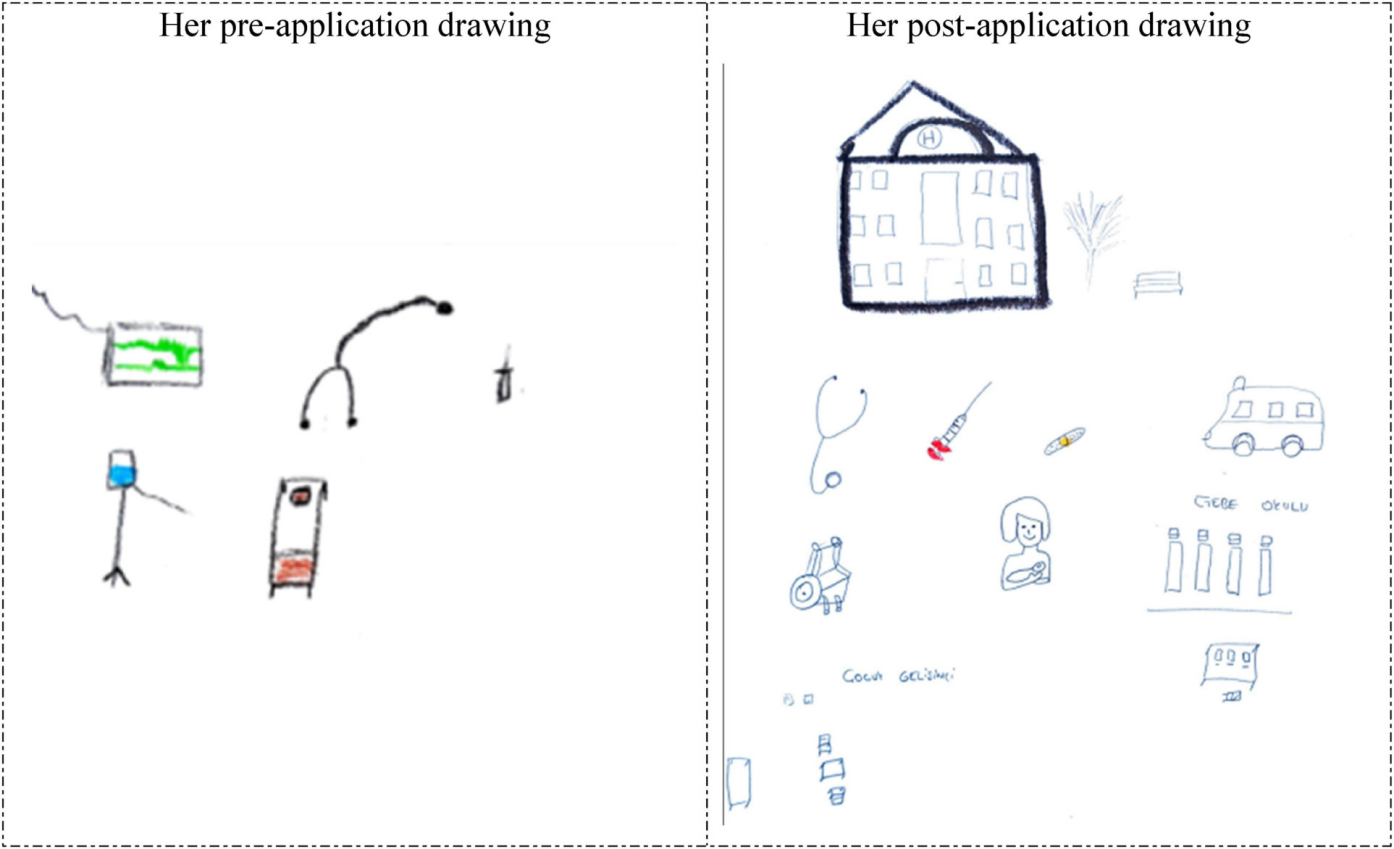
Drawings depicted by the 7th participant before and after hospital practice. The participant who had never been hospitalized, had no illness, considered working in the hospital before the hospital practice and still wanted to work in the hospital. Çocuk gelişimci: Child development; Gebe okulu: Pregnancy school: It is a school where pregnant women are trained in childbirth and baby care.

In her pre-application drawing in Figure 9, the 7th participant depicted medicine-related things such as a stethoscope, syringe, patient bed, intravenous drip, and monitor, all independently of each other. In her post-application drawing, she depicted a hospital building, an ambulance, and a wheelchair, in addition to the things in her pre-application drawing, and her observations from her practical experience. The inclusion of the physical environment of a pregnancy school, a mother who newly gave a birth, and a child development specialist's workspace in the drawing suggests that the participant had a broader perspective on healthcare services (Figure 9).

**Figure 10:**
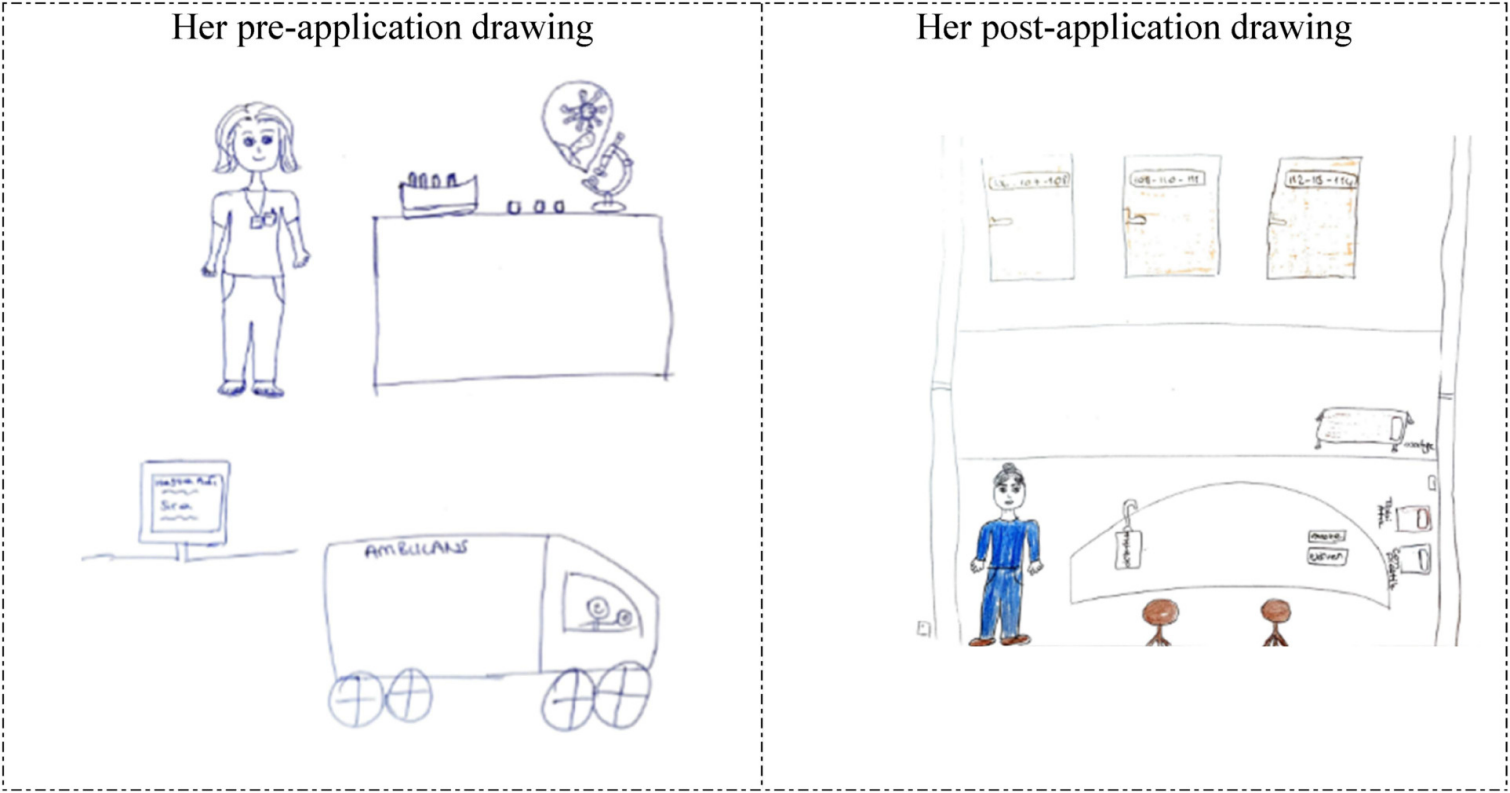
Drawings depicted by the 27th participant before and after hospital practice. The participant who was previously hospitalized for 7 days, and who did not consider working in the hospital before and after the hospital practice. Ambulans: Ambulance; Maske: Mask; Eldiven: Gloves; Tıbbi atık: Medical waste.

In her drawings in Figure 10, the 27th participant who had never considered working in a hospital depicted pre- and post-treatment case. In her pre-application drawing, she drew an ambulance reminiscent of a hospital, as well as locations related to the faculty drama room. In her post-application drawing, she directly depicted an inpatient ward but did not include the child development specialist. From this perspective, she did not view the child development specialist as part of the healthcare team. Her preference for not working in a hospital may have stemmed from her past negative personal experiences, which cannot be elicited through direct questions (Figure 10).

**Figure 11:**
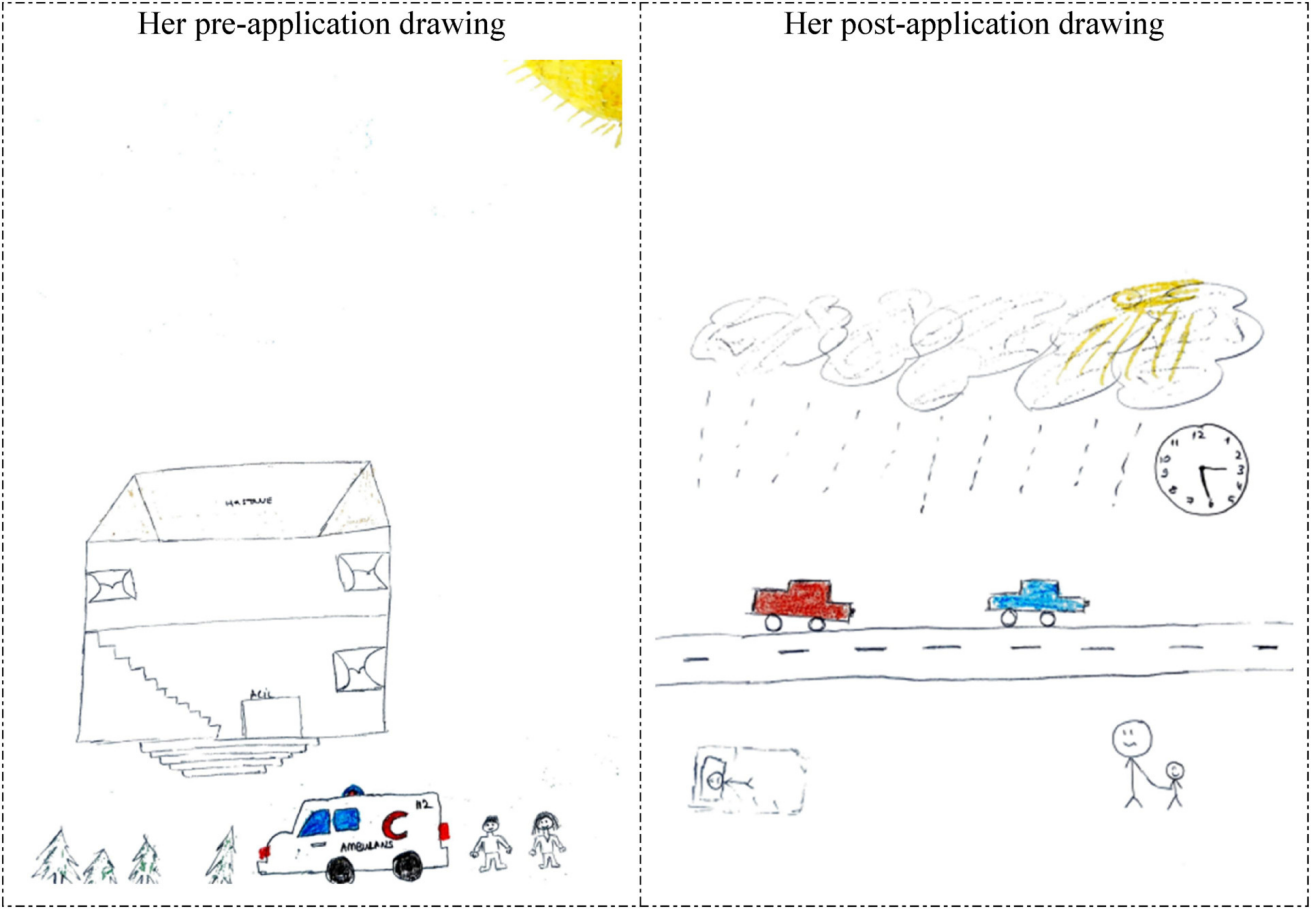
Drawings depicted by the 6th participant before and after hospital practice. The participant who previously accompanied a patient in the hospital, considered working in the hospital before the hospital practice and still wanted to work in the hospital. Hastane: Hospital; Acil service: Emergency service; Ambulans: Ambulance; 112: Emergency call center.

In her pre-application drawing in Figure 11, the 6th participant depicted a hospital where she directly experienced her tasks. This inference stems from the fact that the drawing directly includes the name of the hospital (its name was hidden due to confidentiality principles). In her post-application drawing, she reflected a scenario. She depicted a worried father and a happy child who were walking towards the hospital on a busy street on a rainy day. The inclusion of a clock in the drawing suggests that the event in the drawing corresponds to a memory etched in her mind, distinct in time and place. The drawing also demonstrates that humans are socio-emotional beings. Although she participated in a hospital application for one semester, her personal experience is believed to have played a more dominant role in shaping her perception of the concept of the hospital than the practical experience itself.

## Conclusion and recommendations

Child development specialists working in hospitals contribute to the developmental processes of both typically developing children and children with developmental delays. They also support children's adaptation to the hospital environment, prepare them for surgery, provide education and support to patients and their families, and conduct developmental activities in playrooms, bedside settings, and hospital classrooms (11). According to the results of the present study, the participating child development students’ pre-application drawings demonstrated that they did not perceive the hospital environment as a place where they could practice their profession. However, a significant change was observed in their perceptions in their post-application drawings. Their pre-application drawings were dominated by themes of *a patient bed, intravenous drip, basic medical supplies such as syringes and stethoscopes, and the hospital interior*, the prevalence of these themes decreased in their post-application drawings, and the themes were more evenly distributed, suggesting that their perceptions of the hospital environment diversified and their perspectives broadened. However, although the hospital is a professionally important workspace for child development specialists, the participating students were less inclined to include child development specialists in the hospital environment in their drawings even after the application. The fact that only four participants (13.3%) included a child development specialist and/or their work environment in their post-application drawings suggests that their perception of the professional role within the hospital context is not sufficiently developed. They had prior experiences with the hospital environment in various ways prior to the course (e.g., family members’ health problems or their own hospitalizations). It is believed that their perceptions of hospitals prior to the course may have been shaped by their own personal experiences. These experiences’ being particularly negative may have prevented them from establishing a connection between the hospital environment and their professions. Data from their post-application drawings indicate that the participants did not sufficiently consider themselves as health professionals.

A study examined the impact of summer internship practices on the professional competence perceptions of Child Development students. It was found that internships in kindergartens and preschools influence students’ perceptions of professional competence, whereas this influence was minimal for students interning in hospitals and rehabilitation centers (12). Another study also found that Child Development specialists working in hospitals had a positive perception of their profession; however, their perception of the working environment was negative (6). In addition, a study conducted with nursing students reported that hospital practice positively influenced their perception of clinical preparation (13). Similarly, a positive effect of hospital practice was observed in the present study in Child Development students.

In another study (14) conducted on Child Development Department students, it was found that health awareness increases with higher levels of education. This study was also conducted with third-year students in the spring semester, and it is considered that it might have contributed to the development of students’ perspectives on hospitals.

By the end of their studies, students should perceive the hospital environment from a professional perspective. Indeed, psychosocial support for hospitalized children and their parents was found to be effective in reducing anxiety (15). Accordingly, hospitals are one of the professional settings for Child Development specialists, so greater emphasis is considered necessary for hospital practice. Child development students’ acceptance of the hospital environment should be supported by strengthening their knowledge of hospitals, illnesses, and the healthcare system as members of the healthcare team. In line with this, findings from a study indicate that the majority of healthcare professionals believe that a child development specialist should be employed in every hospital for both hospitalized and healthy children (16).

Within this context, it is recommended that the theoretical content of the Hospital and Child I course be further enriched with topics on hospitals, illnesses, and healthcare services to better familiarize students with the hospital environment. This course also includes field trips to hospitals, family health centers, and especially healthcare institutions where practical training is not possible. Furthermore, increasing the number of weekly hospital practice days, as well as the inclusion of summer internship practice, is considered to be beneficial.

## Data Availability

The raw data supporting the conclusions of this article will be made available by the authors, without undue reservation.
